# 

**Published:** 1899-04-01

**Authors:** 


					I siHj nun
Ai'Rir- ist, i?ay. r i
CADBl - atf* U W I /5ADBURYS
COCOA " 1L JTA JEL" / iyy cocoa %
~ f ?? ?<UGS OR ALKALIES USED.
\r
'nmCH Hospital
S^CLAScorv Western Intirmary
No. 6E3. Vol. XXVT. CSSS#)
Saturday, vVrmTi 1st, 1899.
r Subscription Terms,"1 pRICE TWOPENCE.
S00 p. 4. J

				

